# Monitoring Incidence of COVID-19 Cases, Hospitalizations, and Deaths, by Vaccination Status — 13 U.S. Jurisdictions, April 4–July 17, 2021

**DOI:** 10.15585/mmwr.mm7037e1

**Published:** 2021-09-17

**Authors:** Heather M. Scobie, Amelia G. Johnson, Amitabh B. Suthar, Rachel Severson, Nisha B. Alden, Sharon Balter, Daniel Bertolino, David Blythe, Shane Brady, Betsy Cadwell, Iris Cheng, Sherri Davidson, Janelle Delgadillo, Katelynn Devinney, Jeff Duchin, Monique Duwell, Rebecca Fisher, Aaron Fleischauer, Ashley Grant, Jennifer Griffin, Meredith Haddix, Julie Hand, Matt Hanson, Eric Hawkins, Rachel K. Herlihy, Liam Hicks, Corinne Holtzman, Mikhail Hoskins, Judie Hyun, Ramandeep Kaur, Meagan Kay, Holly Kidrowski, Curi Kim, Kenneth Komatsu, Kiersten Kugeler, Melissa Lewis, B. Casey Lyons, Shelby Lyons, Ruth Lynfield, Keegan McCaffrey, Chelsea McMullen, Lauren Milroy, Stephanie Meyer, Leisha Nolen, Monita R. Patel, Sargis Pogosjans, Heather E. Reese, Amy Saupe, Jessica Sell, Theresa Sokol, Daniel Sosin, Emma Stanislawski, Kelly Stevens, Hailey Vest, Kelly White, Erica Wilson, Adam MacNeil, Matthew D. Ritchey, Benjamin J. Silk

**Affiliations:** ^1^Epidemiology Task Force, CDC COVID-19 Response Team; ^2^Data Analytics and Visualization Task Force, CDC COVID-19 Response Team; ^3^Colorado Department of Public Health and Environment; ^4^Acute Communicable Disease Control Program, Los Angeles County Department of Public Health, California; ^5^New York City Department of Health and Mental Hygiene, New York; ^6^Maryland Department of Health; ^7^Arizona Department of Health Services; ^8^Alabama Department of Health; ^9^Utah Department of Health; ^10^Public Health – Seattle & King County, Washington; ^11^North Carolina Department of Health and Human Services; ^12^Louisiana Department of Health; ^13^Indiana State Department of Health; ^14^Minnesota Department of Health; ^15^New Mexico Department of Health.

COVID-19 vaccine breakthrough infection surveillance helps monitor trends in disease incidence and severe outcomes in fully vaccinated persons, including the impact of the highly transmissible B.1.617.2 (Delta) variant of SARS-CoV-2, the virus that causes COVID-19. Reported COVID-19 cases, hospitalizations, and deaths occurring among persons aged ≥18 years during April 4–July 17, 2021, were analyzed by vaccination status across 13 U.S. jurisdictions that routinely linked case surveillance and immunization registry data. Averaged weekly, age-standardized incidence rate ratios (IRRs) for cases among persons who were not fully vaccinated compared with those among fully vaccinated persons decreased from 11.1 (95% confidence interval [CI] = 7.8–15.8) to 4.6 (95% CI = 2.5–8.5) between two periods when prevalence of the Delta variant was lower (<50% of sequenced isolates; April 4–June 19) and higher (≥50%; June 20–July 17), and IRRs for hospitalizations and deaths decreased between the same two periods, from 13.3 (95% CI = 11.3–15.6) to 10.4 (95% CI = 8.1–13.3) and from 16.6 (95% CI = 13.5–20.4) to 11.3 (95% CI = 9.1–13.9). Findings were consistent with a potential decline in vaccine protection against confirmed SARS-CoV-2 infection and continued strong protection against COVID-19–associated hospitalization and death. Getting vaccinated protects against severe illness from COVID-19, including the Delta variant, and monitoring COVID-19 incidence by vaccination status might provide early signals of changes in vaccine-related protection that can be confirmed through well-controlled vaccine effectiveness (VE) studies. 

Two surveillance indicators that potentially can be used to monitor and describe vaccine breakthrough COVID-19 cases and severe outcomes are the percentage of vaccinated persons among cases (PVC) and an IRR between unvaccinated and vaccinated patients. PVC increases with increasing vaccination coverage or decreasing VE (*1*,*2*), complicating interpretation of this metric. IRRs are more stable, directly related to VE, and easier to communicate publicly in terms of vaccine impact (*2*). Most jurisdictions focus on assessing COVID-19 outcomes in fully vaccinated persons (≥14 days after completion of all recommended doses of an FDA-authorized COVID-19 vaccine) and have readily implemented comparisons to not fully vaccinated persons, including persons who are partially vaccinated (<14 days since completing the primary series or did not complete the series) or unvaccinated (did not receive any COVID-19 vaccine); some jurisdictions also monitor trends in partially vaccinated persons.

Aggregate weekly numbers of COVID-19 cases and COVID-19–associated hospitalizations and deaths among persons aged ≥18 years with specimen collection dates during April 4–July 17, 2021, were analyzed by age group (18–49, 50–64, and ≥65 years) and vaccination status across 13 public health jurisdictions.* All participating jurisdictions had established processes for linking case surveillance and vaccination data from state/local immunization registries; this method usually assumes that cases among persons not matched to the registry are among unvaccinated persons. Eleven jurisdictions provided hospitalization data, and all submitted mortality data. Standard definitions were used for 1) COVID-19 cases,^†^ 2) COVID-19 cases in fully vaccinated or not fully vaccinated persons,^§^ 3) COVID-19–associated hospitalizations,^¶^ and 4) COVID-19–associated deaths,** with specimen collection dates used as time points.

Two analysis periods, April 4–June 19 and June 20–July 17, were designated, based on weeks with <50% or ≥50% weighted prevalence of the SARS-CoV-2 Delta variant for the 13 jurisdictions.^††^ The percentages of total cases, hospitalizations, and deaths by vaccination status were calculated for each period and age group. The expected PVC was assessed using the formula: PVC = [PPV–(PPV*VE)]/[1–(PPV*VE)], where PPV is the proportion of the population vaccinated, or vaccination coverage (*1*). PVC was calculated using VE estimates of 80%, 90%, and 95%. Vaccination coverage was estimated by age group using the sum of fully vaccinated persons divided by the 2019 U.S. intercensal population estimates.^§§^ Weekly age-specific incidences by vaccination status were calculated as the number of cases, hospitalizations, or deaths divided by the number of persons either fully vaccinated or not fully vaccinated (obtained by subtracting the number of fully vaccinated persons from total population estimates). Average weekly incidence in each period was age standardized using the 2000 U.S. Census standard population.^¶¶^ IRRs were calculated by dividing the incidence among persons not fully vaccinated by that among fully vaccinated persons; 95% CIs were calculated to account for variation in weekly rates. To aid interpretation of changes in IRRs, age-standardized crude VE was estimated as (1 – [incidence in vaccinated/incidence in unvaccinated]). A sensitivity analysis examined the impact of excluding partially vaccinated persons from IRRs using data available from nine jurisdictions. SAS (version 9.4; SAS Institute) and R (version 4.0.3; R Foundation) were used to conduct all analyses. This activity was reviewed by CDC and was conducted consistent with applicable federal law and CDC policy.***

During April 4–July 17, a total of 569,142 (92%) COVID-19 cases, 34,972 (92%) hospitalizations, and 6,132 (91%) COVID-19–associated deaths were reported among persons not fully vaccinated, and 46,312 (8%) cases, 2,976 (8%) hospitalizations, and 616 (9%) deaths were reported among fully vaccinated persons in the 13 jurisdictions (Table). The weekly prevalence of the SARS-CoV-2 Delta variant increased from <1% to 90% during April 4–July 17. Full vaccination coverage increased from 19% to 54%; in the final week, coverage ranged by age group from 45% (in persons aged 18–49 years) to 73% (≥65 years).

**TABLE:** Numbers, percentages, incidence rates, and incidence rate ratios * (in not fully vaccinated versus fully vaccinated persons) of COVID-19 cases, hospitalizations,**^†^** and deaths,**^§^** by age group and vaccination status**^¶^** — 13 U.S. jurisdictions,** April 4–June 19 and June 20–July 17, 2021**^††^**

Age group, yrs	Cases		Hospitalizations		Deaths	
	Not fully vaccinated	Fully vaccinated	Not fully vaccinated	Fully vaccinated	Not fully vaccinated	Fully vaccinated
**Totals**	569,142 (92)	46,312 (8)	34,972 (92)	2,976 (8)	6,132 (91)	616 (9)
**April 4–June 19**						
**Total no. (% of total)**						
18–49	331,151 (97)	10,346 (3)	10,526 (97)	295 (3)	609 (99)	7 (1)
50–64	93,474 (94)	5,850 (6)	9,158 (95)	444 (5)	1,380 (96)	58 (4)
≥65	42,884 (85)	7,307 (15)	9,199 (88)	1,286 (12)	3,137 (90)	363 (10)
All ages	467,509 (95)	23,503 (5)	28,883 (93)	2,025 (7)	5,126 (92)	428 (8)
**Average weekly incidence (events per 100,000 population)**						
18–49	122.4	10.9	4.6	0.3	0.2	0.0
50–64	98.3	9.1	11.4	0.8	1.5	0.1
≥65	91.6	8.5	22.8	1.8	6.7	0.4
All ages (crude)	113.4	9.6	8.3	1.0	1.2	0.2
All ages (age standardized)	112.3	10.1	9.1	0.7	1.6	0.1
**Average weekly IRR (95% CI)**						
18–49	11.3 (6.7–18.8)		13.4 (9.5–18.8)		30.7 (11.5–81.5)	
50–64	10.8 (7.4–15.7)		14.0 (11.2–17.6)		16.0 (11.2–22.8)	
≥65	10.7 (8.3–13.9)		12.8 (10.0–16.4)		15.8 (12.2–20.4)	
All ages (crude)	11.8 (8.1–17.2)		8.7 (6.4–11.8)		7.1 (4.9–10.2)	
All ages (age standardized)	11.1 (7.8–15.8)		13.3 (11.3–15.6)		16.6 (13.5–20.4)	
**June 20–July 17**						
**Total no. (% of total)**						
18–49	76,237 (85)	13,030 (15)	2,666 (95)	146 (5)	155 (96)	7 (4)
50–64	17,303 (77)	5,027 (23)	1,755 (88)	234 (12)	290 (93)	23 (7)
≥65	8,093 (63)	4,752 (37)	1,668 (74)	571 (26)	561 (78)	158 (22)
All ages	101,633 (82)	22,809 (18)	6,089 (86)	951 (14)	1,006 (84)	188 (16)
**Average weekly incidence (per 100,000 population)**						
18–49	101.9	22.4	4.3	0.3	0.2	0.0
50–64	72.4	14.8	8.7	0.8	1.2	0.1
≥65	61.8	13.6	14.7	1.9	4.3	0.5
All ages (crude)	90.9	17.9	6.5	0.9	0.9	0.1
All ages (age standardized)	89.1	19.4	7.0	0.7	1.1	0.1
**Average weekly IRR (95% CI)**						
18–49	4.5 (2.0–10.4)		15.2 (10.7–21.6)		17.2 (9.4–31.7)	
50–64	4.9 (2.4–10.1)		10.9 (6.9–17.2)		17.9 (10.6–30.3)	
≥65	4.6 (2.4–8.7)		7.6 (5.2–9.6)		9.6 (7.4–10.8)	
All ages (crude)	5.1 (2.3–11.1)		7.6 (5.1–11.3)		6.1 (4.7–8.0)	
All ages (age standardized)	4.6 (2.5–8.5)		10.4 (8.1–13.3)		11.3 (9.1–13.9)	

**Abbreviations:** CI = confidence interval; FDA = Food and Drug Administration; IRR = incidence rate ratio.

* Average weekly incidence rates and rate ratios are provided by age group and overall, including crude values and values standardized by age, according to the enumerated 2000 U.S. Census age distribution.

^†^ To ascertain COVID-19–associated hospitalizations, two jurisdictions relied upon case investigations; seven jurisdictions relied upon hospital records; two jurisdictions relied upon both case investigations and hospital records; and two did not submit hospitalization data. Four jurisdictions reported hospitalizations only when COVID-19 was the cause, and seven reported COVID-19 cases in persons hospitalized for any cause.

^§^ To ascertain COVID-19–associated deaths, eight jurisdictions relied upon vital records; and five jurisdictions relied upon a combination of vital records and provider reporting (two), case investigations and vital records (two), and provider reporting, case investigations, and vital records (one). Eleven jurisdictions provided deaths with COVID-19 as a cause; one provided all deaths that occurred within 30 days of becoming a case (without confirming cause); and one provided deaths confirmed with COVID-19 as a cause or within 60 days of positive specimen collection.

^¶^ Fully vaccinated persons are those who are ≥14 days postcompletion of the primary series of an FDA-authorized COVID-19 vaccine. Not fully vaccinated persons are those who did not receive an FDA-authorized COVID-19 vaccine or who received vaccine but are not yet considered fully vaccinated.

** Alabama, Arizona, Colorado, Indiana, Los Angeles County (California), Louisiana, Maryland, Minnesota, New Mexico, New York City (New York), North Carolina, Seattle/King County (Washington), and Utah.

^††^ Two analysis periods, April 4–June 19 and June 20–July 17, were designated based on the threshold week when the weighted percentage of lineages from whole-genome sequencing results submitted to or performed by CDC reached 50% for the SARS-CoV-2 B.1.617.2 (Delta) variant across the 13 jurisdictions.

During April 4–June 19, fully vaccinated persons accounted for 5% of cases, 7% of hospitalizations, and 8% of deaths overall; these percentages were higher during June 20–July 17 (18%, 14%, and 16%, respectively). Using the reported 37% vaccination coverage for the 13 jurisdictions during April 4–June 19 and an assumption of 90% VE, vaccinated persons would have been expected to account for 6% of cases (close to the 5% observed). With 53% coverage reported during June 20–July 17, vaccinated persons were expected to account for 10% of cases at a constant VE of 90%; the observed 18% would have been expected at a lower VE of 80%.

Averaged weekly, age-standardized rates (events per 100,000 persons) were higher among persons not fully vaccinated than among fully vaccinated persons for reported cases (112.3 versus 10.1), hospitalizations (9.1 versus 0.7), and deaths (1.6 versus 0.1) during April 4–June 19, as well as during June 20–July 17 (89.1 versus 19.4; 7.0 versus 0.7; 1.1 versus 0.1, respectively). Higher hospitalization and death rates were observed in older age groups, regardless of vaccination status, resulting in a larger impact of age-standardization on overall incidence for these outcomes.

Within each age group, the percentage of vaccinated persons among cases, hospitalizations, and deaths increased with increasing vaccination coverage (Figure 1). As the prevalence of SARS-CoV-2 Delta variant surpassed 50%, the percentage of vaccinated persons among cases in each age group increased at rates corresponding to benchmarks for lower VE (i.e., from approximately 90% to <80%). Increases in the percentages of vaccinated persons aged ≥65 years among COVID-19–associated hospitalizations and deaths also appeared higher than expected. During June 20–July 17, age-standardized rates of cases, hospitalizations, and deaths among persons not fully vaccinated increased weekly; among fully vaccinated persons, case rates increased, but rates of hospitalizations and deaths remained largely unchanged (Figure 2).

**FIGURE 1:**
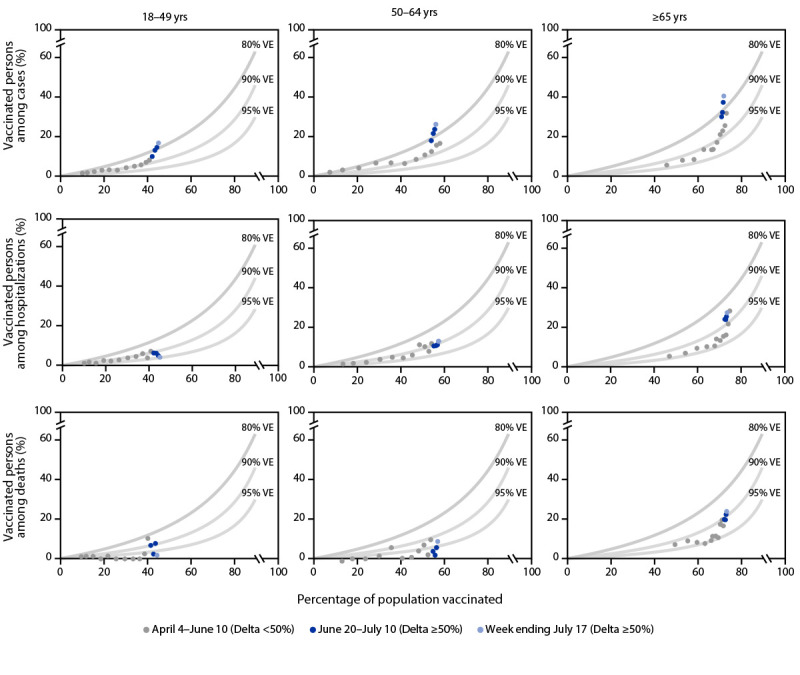
Observed versus expected percentage of fully vaccinated persons among COVID-19 cases, hospitalizations, and deaths based on population vaccination coverage* and assumed 80%–95% vaccine effectiveness,^†^ by week^§^ and age group — 13 U.S. jurisdictions,^¶^ April 4–July 17, 2021 **Abbreviations:** PVC = percentage of vaccinated persons occurring among outcomes; PPV = proportion of the population that is vaccinated; VE = vaccine effectiveness. * Vaccination coverage was estimated using the sum of fully vaccinated persons (submitted by the jurisdictions) divided by the combined 2019 U.S. intercensal population estimates by age group. ^†^ The expected PVC, represented by the light gray lines, was assessed using the formula: PVC = [PPV-(PPV*VE)]/1-(PPV*VE), where benchmarks are added at different VE values (80%, 90%, and 95%). Observed values that approach or go above the 80% VE line indicate decreased VE. ^§^ Two analysis periods, April 4–June 19 and June 20–July 17, were designated based on the threshold week when the weighted percentage of lineages from whole-genome sequencing results submitted to or performed by CDC reached 50% for the SARS-CoV-2 B.1.617.2 (Delta) variant across the 13 jurisdictions. Weekly values are plotted, with the two analysis periods and most recent week for the analysis period shown. ^¶^ Alabama, Arizona, Colorado, Indiana, Los Angeles County (California), Louisiana, Maryland, Minnesota, New Mexico, New York City (New York), North Carolina, Seattle/King County (Washington), and Utah.

**FIGURE 2:**
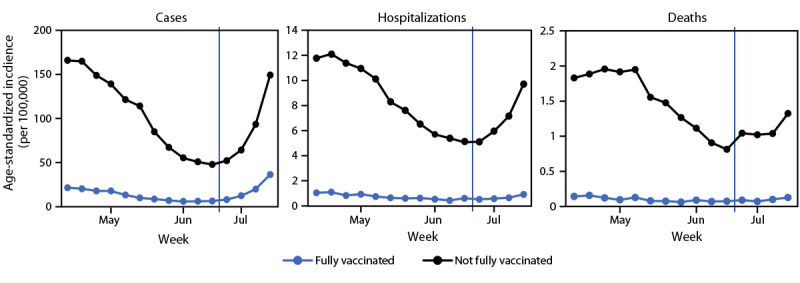
Weekly trends in age-standardized incidence* of COVID-19 cases, hospitalizations,^†^ and deaths,^§^ by vaccination status^¶^ — 13 U.S. jurisdictions,** April 4–July 17, 2021 * Rates are standardized by age, according to the enumerated 2000 U.S. Census age distribution. Blue vertical lines indicate when the B.1.617.2 (Delta) variant reached a threshold of >50%, using weighted estimates for 13 jurisdictions combined. ^†^ To ascertain COVID-19–associated hospitalizations, two jurisdictions relied upon case investigations; seven jurisdictions relied upon hospital records; two jurisdictions relied upon both case investigations and hospital records; and two did not submit hospitalization data. Four jurisdictions reported hospitalizations only where COVID-19 was the cause, and seven reported COVID-19 cases in persons hospitalized for any cause. ^§^ To ascertain COVID-19–associated deaths, eight jurisdictions relied upon vital records, and five jurisdictions relied upon a combination of vital records and provider reporting (two), case investigations and vital records (two), and provider reporting, case investigations, and vital records (one). Eleven jurisdictions provided deaths with COVID-19 as a cause; one provided all deaths that occurred within 30 days of becoming a case (without confirming cause); and one provided deaths confirmed with COVID-19 as a cause or within 60 days of positive specimen collection. ^¶^ Fully vaccinated persons are those who are ≥14 days postcompletion of the primary series of a COVID-19 vaccine with Food and Drug Administration emergency use authorization. Not fully vaccinated persons are those who did not receive a COVID-19 vaccine with Food and Drug Administration emergency use authorization or who received a COVID-19 vaccine but are not yet considered fully vaccinated. ** Alabama, Arizona, Colorado, Indiana, Los Angeles County (California), Louisiana, Maryland, Minnesota, New Mexico, New York City (New York), North Carolina, Seattle/King County (Washington), and Utah.

Age-standardized IRRs for cases in persons not fully vaccinated versus fully vaccinated decreased from 11.1 (95% CI = 7.8–15.8) during April 4–June 19 to 4.6 (95% CI = 2.5–8.5) during June 20–July 17, while IRRs decreased slightly from 13.3 (95% CI = 11.3–15.6) to 10.4 (95% CI = 8.1–13.3) for hospitalizations and from 16.6 (95% CI = 13.5–20.4) to 11.3 (95% CI = 9.1–13.9) for deaths during the same two periods. Persons aged ≥65 years had larger declines in IRRs for hospitalization and death than did younger age groups (Table). The change in age-standardized IRRs for cases between the April 4–June 19 and June 20–July 17 periods represented potential changes in crude VE from 91% to 78% for infection, from 92% to 90% for hospitalization, and from 94% to 91% for death (Supplementary Figure 1, https://stacks.cdc.gov/view/cdc/109531). A sensitivity analysis excluding partially vaccinated persons in nine jurisdictions yielded similar trends but higher IRRs and VE estimates for hospitalizations and deaths (Supplementary Table, https://stacks.cdc.gov/view/cdc/109533). Variability in IRRs was also observed among jurisdictions (Supplementary Figure 2, https://stacks.cdc.gov/view/cdc/109532).

## Discussion

In 13 U.S. jurisdictions, rates of COVID-19 cases, hospitalizations, and deaths were substantially higher in persons not fully vaccinated compared with those in fully vaccinated persons, similar to findings in other reports (*2*,*3*). After the week of June 20, 2021, when the SARS-CoV-2 Delta variant became predominant, the percentage of fully vaccinated persons among cases increased more than expected for the given vaccination coverage and a constant VE. The IRR for cases among persons not fully vaccinated versus fully vaccinated decreased substantially; IRRs for hospitalizations and deaths changed less overall, but moderately among adults aged ≥65 years. Findings from this crude analysis of surveillance data are consistent with recent studies reporting decreased VE against confirmed infection but not hospitalization or death, during a period of Delta variant predominance and potential waning of vaccine-induced population immunity (*4*–*6*).^†††^

The findings in this report are subject to at least five limitations. First, combining unvaccinated and partially vaccinated persons resulted in lower IRR and VE estimates. Second, variable linkage of case surveillance, vaccination, hospitalization, and mortality data might have resulted in misclassifications that could influence IRR estimates; no substantial differences in ascertainment of outcomes by vaccination status were noted in jurisdictions that were able to assess this. Lags in reporting of deaths might have affected the second period differentially. Third, this was an ecological study in which IRRs lacked multivariable adjustments and causality could not be assessed (i.e., possible differences in testing or behaviors in vaccinated and unvaccinated persons). VE is being assessed through ongoing controlled studies. Fourth, the period when the SARS-CoV-2 Delta variant reached ≥50% overall prevalence was assumed to be the first week when most cases were infected with the Delta variant, but the week varied by jurisdiction. Finally, the data assessed from 13 jurisdictions accounted for 25% of the U.S. population, and therefore might not be generalizable.

Monitoring COVID-19 outcomes in populations over time by vaccination status is facilitated through reliable linkage of COVID-19 case surveillance and vaccination data. However, interpreting state-level variation by week might be challenging, especially for severe outcomes with small numbers. The framework used in this analysis allows for comparisons of observed IRRs and percentages of vaccinated cases, hospitalizations, and deaths to expected values. The data might be helpful in communicating the real-time impact of vaccines (e.g., persons not fully vaccinated having >10 times higher COVID-19 mortality risk) and guiding prevention strategies, such as vaccination and nonpharmacologic interventions.

**Summary**

**What is already known about this topic?**

The incidence of SARS-CoV-2 infection, hospitalization, and death is higher in unvaccinated than vaccinated persons, and the incidence rate ratios are related to vaccine effectiveness.

**What is added by this report?**

Across 13 U.S. jurisdictions, incidence rate ratios for hospitalization and death changed relatively little after the SARS-CoV-2 B.1.617.2 (Delta) variant reached predominance, suggesting high, continued vaccine effectiveness against severe COVID-19. Case IRRs decreased, suggesting reduced vaccine effectiveness for prevention of SARS-CoV-2 infections.

**What are the implications for public health practice?**

Getting vaccinated protects against severe illness from COVID-19, including the Delta variant. Monitoring COVID-19 incidence by vaccination status might provide early signals of potential changes in vaccine effectiveness that can be confirmed through robust controlled studies.

## Footnotes

* Alabama, Arizona, Colorado, Indiana, Los Angeles County (California), Louisiana, Maryland, Minnesota, New Mexico, New York City (New York), North Carolina, Seattle/King County (Washington), and Utah. Portions of the population in Colorado (49%), Minnesota (55%), New Mexico (61%), and Utah (35%) and the whole population of Maryland are included as part of the COVID-19–Associated Hospitalization Surveillance Network (COVID-NET). https://www.cdc.gov/coronavirus/2019-ncov/covid-data/covid-net/purpose-methods.html

† A COVID-19 case (confirmed or probable) was defined as the detection of SARS-CoV-2 RNA or antigen in a respiratory specimen collected from a person aged ≥18 years per the Council of State and Territorial Epidemiologists’ update to the standardized surveillance case definition and national notification for 2019 novel coronavirus disease (COVID-19) (21-ID-01): https://cdn.ymaws.com/www.cste.org/resource/resmgr/ps/ps2021/21-ID-01_COVID-19.pdf. Known cases of SARS-CoV-2 reinfection were excluded.

§ A COVID-19 case in a fully vaccinated person (i.e., a breakthrough infection) occurred ≥14 days after completion of the primary series of a COVID-19 vaccine with Food and Drug Administration emergency use authorization per CDC’s definition of a fully vaccinated person (https://www.cdc.gov/coronavirus/2019-ncov/vaccines/fully-vaccinated-guidance.html). Fully vaccinated persons were those who received a Pfizer-BioNTech or Moderna mRNA vaccine (92%) or the Janssen (Johnson & Johnson) vaccine (8%). A COVID-19 case in a person who was not fully vaccinated occurred when the person did not receive an FDA-authorized COVID-19 vaccine or received less than a complete primary series or if <14 days had elapsed since completing a primary series of an FDA-authorized vaccine before the specimen collection date. This analysis represents the combined impact of the Pfizer-BioNTech, Moderna, and Janssen vaccines, which had different clinical efficacies against confirmed infection (95%, 94%, and 67%, respectively). Information on different FDA-authorized and approved COVID-19 vaccine products, including clinical efficacy is available at https://www.cdc.gov/coronavirus/2019-ncov/vaccines/different-vaccines.html.

¶ A COVID-19–associated hospitalization was a COVID-19 case in a person hospitalized within 14 days of a SARS-CoV-2 positive specimen collection ordered by a health care professional. To ascertain COVID-19–associated hospitalization status, two jurisdictions relied upon case investigations, seven relied upon hospital records, two relied upon both case investigations and hospital records, and two did not submit hospitalization data. Four jurisdictions reported hospitalizations only when COVID-19 was the cause, and seven reported COVID-19 cases in persons hospitalized for any cause.

** A COVID-19–associated death occurred in a person with a documented COVID-19 diagnosis who died, and whose death local health authorities reviewed to make a determination using vital records, public health investigation, or other data sources. To ascertain COVID-19–associated death status, eight jurisdictions relied upon vital records, and five relied upon a combination of vital records and provider reporting (two), case investigations and vital records (two), and provider reporting, case investigations, and vital records (one). Eleven jurisdictions provided deaths with COVID-19 as a cause, one provided all deaths that occurred within 30 days of attaining case status (without confirming cause), and one provided deaths confirmed with COVID-19 as a cause or within 60 days of a positive specimen collection.

†† SARS-CoV-2 variant weighted prevalence estimates are based on whole-genome sequencing results submitted to or performed by CDC (https://covid.cdc.gov/covid-data-tracker/#variant-proportions). By jurisdiction, the SARS-CoV-2 Delta variant surpassed ≥50% prevalence, using unweighted estimates, in the weeks ending June 12, 2021 (one); June 19, 2021 (one); June 26, 2021 (two); and July 3, 2021 (nine).

§§
https://www.census.gov/programs-surveys/popest/data/tables.2019.html

¶¶ To improve comparability of age-standardized rates across data systems, in 1998, the Secretary of the U.S. Department of Health and Human Services (HHS) issued a policy directing all HHS agencies to use the 2000 Standard Population to age standardize death rates. https://www.cdc.gov/nchs/data/statnt/statnt20.pdf

*** 45 C.F.R. part 46.102(l)(2), 21 C.F.R. part 56; 42 U.S.C. Sect.241(d); 5 U.S.C.0 Sect.552a; 44 U.S.C. Sect. 3501 et seq.

††† https://www.medrxiv.org/content/10.1101/2021.08.11.21261885v1; https://papers.ssrn.com/sol3/papers.cfm?abstract_id=3909743; https://www.medrxiv.org/content/10.1101/2021.08.06.21261707v3
